# Heutagogy: A Comprehensive Review of Self-Determined Learning in Contemporary Education

**DOI:** 10.7759/cureus.89731

**Published:** 2025-08-10

**Authors:** Raju Panta

**Affiliations:** 1 Physiology and Pathology/Physiology, Burrell College of Osteopathic Medicine, Melbourne, USA

**Keywords:** active learning, andragogy, heutagogy, independent learning, learning approaches, lifelong learning, self-determined learning, self-directed learning, technology in learning

## Abstract

Heutagogy, or self-determined learning, is an emerging educational framework that emphasizes learner autonomy, capability development, and reflective practice. Unlike pedagogy and andragogy, heutagogy places learners in control of their learning goals, strategies, and outcomes, fostering adaptability and lifelong learning. As digital and AI-enhanced tools reshape educational environments, heutagogy offers a promising model for fostering adaptability and lifelong learning. This review explores heutagogy not merely as an extension of pedagogy and andragogy, but as a distinct and evolving framework suited to the demands of 21st-century education. A structured literature review was conducted using four databases: PubMed, Medline, PubMed Central, and Google Scholar. The search strategy used keywords such as andragogy, heutagogy, self-determined learning, lifelong learning, active learning, self-directed learning, independent learning, technology in learning, and learning approaches. Inclusion criteria focused on peer-reviewed articles published between 2000 and 2025 that addressed heutagogical theory, implementation, and outcomes. Exclusion criteria included non-English publications, opinion pieces, and articles lacking empirical or theoretical grounding. A total of 22 articles met the inclusion criteria and were analyzed using a narrative thematic approach. Thematic analysis identified four key domains: (1) learner agency and self-reflection; (2) double-loop learning and capability development; (3) integration of digital and AI tools to support autonomous learning; and (4) institutional and faculty-level challenges in implementing heutagogical models. The review revealed consistent support for heutagogy’s potential to enhance learner engagement and adaptability, though implementation varied across contexts. This review highlights heutagogy’s relevance in contemporary education, particularly in digitally mediated learning environments. While the framework shows promise for cultivating self-directed learners, successful adoption requires institutional commitment, faculty training, and further empirical validation. Heutagogy stands as a transformative approach for preparing learners to navigate complexity and change.

## Introduction and background

The evolution of educational theory has long been marked by shifts from teacher-centered pedagogy to learner-centered andragogy, culminating in heutagogy, a model that emphasizes learner-determined pathways. Pedagogy, traditionally associated with children’s education, emphasizes passive learning, where the teacher controls content, pace, and assessment, and learners absorb information with limited autonomy. Andragogy, introduced by Malcolm Knowles, shifted the focus to adult learners, recognizing their capacity for self-direction and valuing experiential learning. This approach fosters active engagement through collaboration and problem-solving. Heutagogy, or self-determined learning, represents a further progression. It empowers learners not only to direct their learning but also to define what and how they learn, emphasizing capability development, double-loop learning, and adaptability in complex environments. In heutagogical settings, learners are active agents-reflective, autonomous, and capable of navigating novel situations. This framework is particularly relevant in digitally mediated education, where rapid change demands flexible, lifelong learning strategies [1,2]. Double-loop learning refers to a deeper form of learning where individuals not only correct actions based on feedback (single-loop learning) but also critically examine and revise the underlying assumptions, values, and goals that guide those actions. This reflective process aligns closely with heutagogy’s emphasis on learner autonomy and adaptability in complex environments. While pedagogy and andragogy focus on structured knowledge acquisition and self-directed learning, respectively, heutagogy introduces a paradigm where learners define their own learning objectives, strategies, and assessments. This shift is particularly relevant in the digital age, where learners have unprecedented access to tools that support autonomy and personalized learning. By embracing its principles and the evolving role of educators, heutagogy offers a forward-thinking, adaptable approach to education in a rapidly changing world [3]. Several studies have demonstrated that the recent advances in technologies and artificial intelligence support heutagogical learning by enabling learners to create their own content and take control of their learning journey. This learner-centered environment encourages self-directed exploration and personalized learning paths [4-6].

Objective

This article moves beyond a conceptual review to analyze heutagogy through the lens of empirical evidence and structured implementation. We examine how heutagogical principles are applied in health professions education, digital learning environments, and problem-based learning (PBL). Drawing on studies that utilize Self-Determination Theory (SDT), we explore how autonomy-supportive environments enhance learner motivation and outcomes. We also assess the role of digital tools, including AI platforms like ChatGPT, in operationalizing heutagogical practices.

Methods

This review was conducted through a comprehensive and structured search of relevant literature across four major databases: PubMed, Medline, PubMed Central, and Google Scholar. The search strategy in PubMed utilized both Medical Subject Headings (MeSH) and free-text terms to enhance retrieval accuracy. The keywords used in search included: andragogy, heutagogy, self-determined learning, lifelong learning, active learning, self-directed learning, independent learning, technology in learning, and learning approaches. Boolean operators were applied to construct the search query, such as ("heutagogy" OR "self-determined learning") AND ("andragogy" OR "self-directed learning") AND ("lifelong learning" OR "independent learning") AND ("technology in learning" OR "digital learning" OR "AI in education"). The search was limited to English-language publications from 2000 to 2025. Studies published prior to 2000 or in other languages were excluded.

A two-stage screening process was used: first, titles and abstracts were screened for relevance; second, eligible full texts were reviewed based on thematic alignment with heutagogical principles and technology integration.

**Figure 1:**
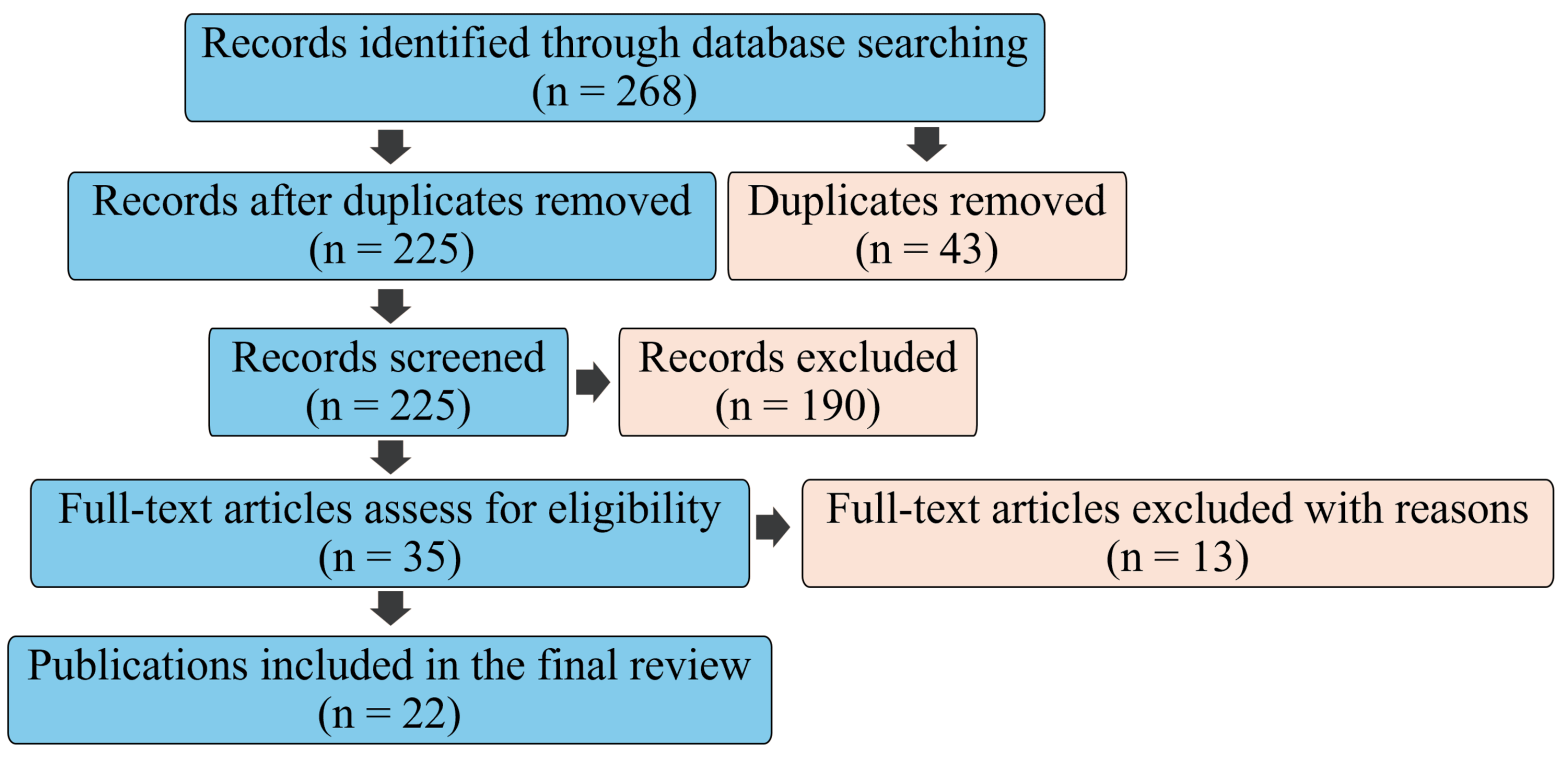
Flow chart of search execution details

The search was executed between April 29 and June 12, 2025, yielding approximately 268 records. After removing 43 duplicates, 225 records remained for title and abstract screening. Of these, 190 were excluded for not meeting the inclusion criteria. The remaining 35 full-text articles were assessed for eligibility, resulting in 22 studies being included in the final review (Figure 1).

Inclusion criteria focused on peer-reviewed articles published between 2000 and 2025 that addressed heutagogical theory, self-determined learning, and the integration of digital or AI tools in educational contexts. Exclusion criteria included non-English publications, opinion pieces, articles lacking empirical or theoretical grounding, and articles published before 2000.

Risk of Bias Assessment

Although the review is primarily narrative, a basic quality appraisal was conducted using a checklist adapted from the Critical Appraisal Skills Programme (CASP) [7]. Each study was evaluated for clarity of aims, methodological rigor, relevance to heutagogical principles, and transparency of findings. Due to the heterogeneity of study designs, a formal risk of bias scoring system was not applied.

Summary Methods

Findings were synthesized using a narrative thematic approach. Key themes, such as learner agency, capability development, double-loop learning, and the role of digital tools, were identified through iterative reading and manual coding of the included studies. No software was used for analysis; instead, themes were grouped based on conceptual alignment with heutagogical principles and SDT.

## Review

Theoretical foundations and analytical frameworks

Heutagogy was first introduced by Stewart Hase and Chris Kenyon in 2000 as an extension of andragogy. While pedagogy is teacher-directed and andragogy is learner-directed, heutagogy is learner-determined. It emphasizes the learner’s agency in deciding what, how, and when to learn [1,8]. The pedagogy-andragogy-heutagogy (PAH) continuum (Table 1) illustrates the progression from teacher-directed to learner-determined education. While pedagogy emphasizes content delivery and teacher control, andragogy introduces learner autonomy and self-direction, and heutagogy extends this by empowering learners to define what and how they learn [1,2,9,10].

**Table 1:** PAH continuum Comparative overview of key attributes in pedagogical, andragogical, and heutagogical learning paradigms. This framework illustrates the progression from instructor-led to learner-determined models, highlighting increased autonomy and capability development. PAH: pedagogy-andragogy-heutagogy

Dimension	Pedagogy (teacher-directed)	Andragogy (self-directed)	Heutagogy (self-determined)
Learning control	Teacher-directed	Learner-directed	Learner-determined
Learning focus	Content delivery	Autonomy and self-direction	Learner agency and capability
Learning loop	Not specified	Single-loop (experiential)	Double-loop (reflection on experience)
Development focus	Knowledge acquisition	Competency development	Capability development
Learning design	Linear	Linear	Nonlinear and flexible
Direction	Instructor-led	Instructor–learner collaboration	Learner-led

This continuum is not strictly linear. Learners may move fluidly between stages depending on context, content, and maturity. Educators are encouraged to adopt a flexible, learner-responsive approach [11].

The theoretical underpinnings of heutagogy are rooted in constructivism, where learners construct knowledge through experience; humanism, which emphasizes personal growth and learner agency; complexity theory, which views learning as emergent and adaptive; and SDT, which explains motivation along a continuum from amotivation to intrinsic motivation [8,12-14].

SDT identifies three basic psychological needs, autonomy, competence, and relatedness, that are essential for intrinsic motivation and well-being. These needs are foundational to heutagogical learning environments. SDT has been widely applied in health professions education to understand how autonomy-supportive environments enhance motivation, engagement, and academic outcomes [14,15].

Principles of heutagogy: empirical insights

The core principles of heutagogy, learner agency, capability development, double-loop learning, non-linear pathways, and metacognition, are consistently supported by empirical research. Learner agency and autonomy promote motivation and engagement when learners choose their own goals and paths [13]. Capability development nurtures adaptability and confidence essential for lifelong learning [16]. Double-loop learning encourages learners to reflect on both outcomes and underlying assumptions, enhancing critical thinking [2]. Non-linear learning has flexible and personalized learning paths and metacognition engages learners in continuous self-assessment and growth [2].

These principles are consistently highlighted in both theoretical discussions [2,3] and empirical studies [17,18]. These principles align with SDT’s emphasis on autonomy, competence, and relatedness as psychological needs that foster intrinsic motivation [19]. Double-loop learning involves not only solving problems but also questioning underlying assumptions and beliefs [20]. This reflective process is central to heutagogical learning. Heutagogy emphasizes learner agency and autonomy, intrinsic motivation, collaborative and networked learning, and integration of digital and AI tools [5].

Structured implementations in practice

Health Professions Education

Recent studies have explored self-determined motivation in digital learning environments. Studies show that heutagogy enhances learner engagement, autonomy, and capability in medical education. A systematic review highlighted that self-determined motivation in medical students is associated with better academic performance, engagement, and well-being. Factors such as feedback, autonomy-supportive climates, and meaningful learning experiences were key determinants [14]. Moroccan nursing students in distance learning environments showed increased motivation when exposed to autonomy-supportive teaching [15]. Similarly, implementing a heutagogical model in a mentored student research project enhanced reflective practice and collaborative learning [16]. Application of SDT to understand the impostor phenomenon (IP) in medical students demonstrated that students with higher controlled motivation and lower satisfaction of basic psychological needs experienced more severe IP symptoms. This highlights the importance of autonomy-supportive environments in reducing psychological distress and fostering self-worth in learners [17].

Digital and Online Learning

Digital platforms are ideal for heutagogical learning. Web 2.0 and social media tools such as blogs, wikis, podcasts, and social networking support heutagogical learning by enabling learner-generated content, collaboration, and reflection. These tools promote active participation and knowledge construction, essential for heutagogical environments [4]. AI tools like ChatGPT, adaptive learning platforms, chatbots, and mobile apps enable learners to access resources, personalize learning, and provide real-time feedback, aligning with heutagogical principles [5,6].

Problem-Based and Blended Learning

An approach of a heutagogical framework for PBL that integrates social media to enhance learner autonomy and cognitive engagement encourages learners to co-create content, choose learning strategies, and reflect on their learning process, which are key aspects of capability development [20]. Facebook has been used effectively to implement heutagogical principles among millennial learners [21]. Similarly, blended learning environments offer flexibility and support non-linear, personalized learning paths, making them ideal for heutagogical practice [9].

Educator and learner readiness

Educator Perspectives

While many educators are unfamiliar with the term "heutagogy," they recognize its core principles, such as self-determination, reflective learning, and learner autonomy. However, concerns remain about its practicality, especially regarding assessment and teacher authority. Teachers who embrace heutagogy often shift from being "sages on the stage" to "guides on the side," facilitating rather than directing learning. This transition requires professional development and a willingness to relinquish control [9].

A study found varied levels of understanding and readiness for implementing heutagogy among lecturers and preservice teachers. While lecturers were generally enthusiastic about its potential to enhance learner autonomy and critical thinking, preservice teachers showed mixed comfort and preparedness for self-directed learning approaches [22].

Learner Perspectives

Studies show that learners with higher self-determined motivation engage more deeply with content, experience greater well-being and satisfaction, perform better academically, and are more likely to pursue lifelong learning. Conversely, amotivation is linked to stress, burnout, and disengagement [14,15,23].

Technology as a catalyst

The integration of Web 2.0 tools into medical education has been shown to increase student motivation and engagement, facilitate collaborative learning, and support reflective practice. These tools align with heutagogical principles by enabling learners to control their learning paths and co-create knowledge [4]. Showcasing ChatGPT as a tool for self-determined learning in medical education highlights its potential to transform teaching by fostering personalized, accessible, and effective learning experiences. Thoughtful and ethical integration of AI like ChatGPT can better prepare learners for the complexities of the modern world [6]. The Fourth Industrial Revolution demands that medical education embrace lifelong, self-determined learning models like heutagogy. By fostering autonomy, reflection, and adaptability and integrating technology, healthcare professionals can continuously “learn, unlearn, and relearn” to stay competent and future-ready [22].

Limitations

While this manuscript explores the intersection of AI tools and heutagogical learning through a comprehensive synthesis of existing literature, several limitations warrant acknowledgment. First, although systematic review methodologies informed the search and selection process, the study remains a narrative review and does not employ formal systematic review protocols such as PRISMA or meta-analytic techniques. As such, the findings may be subject to selection bias and lack the replicability associated with more structured review designs.

Second, the heutagogical framework itself presents conceptual challenges, including its broad definitional boundaries and limited empirical validation across diverse educational contexts. Prior research in this area is still emerging, and many studies rely on anecdotal or theoretical claims rather than robust outcome data.

Finally, while the manuscript discusses the potential of AI tools to support learner autonomy and self-determined learning, it does not provide concrete, actionable strategies for integrating these technologies into heutagogical practice. Future work should aim to bridge this gap by offering implementation models, case studies, or tool-specific guidance to support educators and institutions.

## Conclusions

Heutagogy represents both a continuation and a redefinition of educational practice, building on the foundations of andragogy while introducing transformative principles that align with the demands of digital fluency, lifelong learning, and learner empowerment. The review demonstrates that heutagogy is not only theoretically robust but also practically applicable across diverse educational contexts, particularly in health professions and digital learning environments. Empirical evidence supports its effectiveness in enhancing learner motivation, engagement, and capability. However, successful implementation requires thoughtful integration of technology, professional development for educators, and institutional flexibility. As education continues to evolve in response to technological and societal shifts, heutagogy offers a compelling and necessary framework for fostering autonomy, adaptability, and lifelong learning in the 21st century.
